# Renewable compensation policies and conventional energy investment: A theoretical model

**DOI:** 10.1016/j.heliyon.2024.e33971

**Published:** 2024-07-06

**Authors:** Boris Ortega Moreno

**Affiliations:** SnT, Interdisciplinary Centre for Security, Reliability and Trust, University of Luxembourg, Luxembourg

**Keywords:** Renewable compensation policies, Natural gas, Capacity investment, Cournot duopoly

## Abstract

Many jurisdictions are simultaneously expanding natural gas and renewable capacities, largely supported by renewable compensation policies (RCPs). However, RCPs' impacts on firms' incentives for conventional capacity investment remain unclear. This paper develops a two-stage theoretical model to investigate this interaction within an imperfect competition and uncertain demand context. Firms initially invest in conventional energy capacity, followed by competing to supply electricity from conventional and previously owned renewables. Conventional output is compensated at market prices, but renewable output is subject to two common RCPs: feed-in tariffs (FiT) and feed-in premiums (FiP). The illustrative numerical example shows that increasing the proportion of renewable output compensated by a FiT from 20% to 80% increases the market-level conventional investment by 18%, leading to an increase in consumer surplus but decreasing firms' profits. These results exemplify the unintended effects of RCPs, encouraging the adoption of conventional generation capacity. The model presented in this paper provides a theoretical foundation for understanding the relationship between RCPs and conventional energy capacity investment—critical for carbon-intensive nations transitioning to renewables while maintaining reliable electricity supply through conventional generation.

## 1 Introduction

Renewable compensation policies (RCPs), which encompass feed-in tariffs (FiTs) and feed-in premiums (FiPs),^1^ stand out as highly effective mechanisms for encouraging the adoption of renewable energy sources ([1]; [2]). However, a key area that requires deeper exploration is their influence on conventional capacity investment (e.g., natural gas). This issue gains particular interest in countries with significant carbon-intensive footprints, such as China and India, where investments in natural gas capacity persist alongside the push for renewable energy transition ([3]; [4]; [5]; [6]). Even in the United States, RCPs and renewable portfolio standards^2^ have surprisingly bolstered the expansion of natural gas-fired plants, amplifying investments and consumption of natural gas within the electricity sector ([7]). This continued expansion of natural gas capacity, even in settings when RCPs are implemented to boost renewable energy sources, raises the question about the relationship between these RCPs and conventional capacity investment. So far, this relationship has not been analyzed, which strengthen the relevance of this paper.

This paper studies how two commonly used RCPs, FiT and FiP, interact with investments in conventional energy capacity in a setting with imperfect competition and uncertain demand. The main objective of the paper is to build a theoretical framework to identify a direct channel that relates these RCPs and conventional energy capacity investment. The theoretical framework considers a scenario in which firms own a fixed amount of renewable capacity and can invest in conventional capacity to generate and sell electricity. The analysis relies on a two-stage game theoretical model and a numerical example to complement the main findings.

The main contribution of this study is to be the first to establish a direct connection between RCPs and firms' conventional capacity investment decisions. The insights of the model serve a steppingstone to understand the spillover effects that RCPs have on other markets, such as natural gas capacity investments. By providing insights into the directions of these effects and the key parameters to consider, this model offers valuable guidance for policymakers, which could have a significant impact on the support scheme to the current renewable energy transition. For instance, according to the model, policymakers should rely more on FIP if they want to discourage conventional capacity investment while strengthening renewable energy adoption. Furthermore, the paper contributes to the ongoing efforts to formally model the most economically efficient ways to mitigate climate change and encourage sustainability through renewable support policies in electricity markets.

The main results show an ambiguous effect of RCPs on firms' conventional capacity investment, which depends on the relative size of the renewable capacity owned by the firms. For instance, consider the case where a big firm owns a sufficiently large share of the total renewable energy capacity. In this case, an increase in the proportion of renewable output subject to a FiT increases the optimal conventional capacity investment of the big firm and decreases it for the small firm (i.e., the firm with the lowest share of renewable capacity). Nevertheless, the total conventional capacity invested in the market unambiguously increases when a FiT becomes more dominant. This is because, in every scenario, the positive effect on the big firm is always relatively larger than the negative effect on the firm with the least renewable capacity. Further, the results show that when a FiT becomes more dominant, the equilibrium price is always negatively affected, and the total equilibrium market output increases. The numerical example results show that consumer surplus increases and firms' profits decrease as a FiT compensates more renewable output. These opposing effects lead to an unambiguous decrease in total surplus.

Summing up, RCPs are widely used to boost the adoption of renewable energy sources, but their effects on capacity investment of conventional energy are unknown. These effects are particularly relevant for countries that rely on natural gas to back up their renewable energy transition. This paper aims to identify the effects of RCPs on conventional energy capacity investment by developing a stylized theoretical model in a setting of imperfect competition and uncertain energy demand. The paper's novelty rests on being the first study to establish a direct connection between RCPs and firms' conventional capacity investment decisions. This relationship is of special interest to policymakers seeking to encourage the adoption of renewable energy sources, while maintaining the attractiveness of natural gas capacity investment as a back up option for reliable generation.

The rest of the paper is structured as follows. Section 2 presents the relevant literature related to capacity investment decisions in electricity markets. Section 3 describes the model. Section 4 derives the equilibrium outcomes and discusses the main results. Section 5 presents a numerical example to illustrate the main results of the model. Section 6 dives into the main policy suggestions derived from the model. Finally, Section 7 concludes.

## 2 Literature review

This study contributes to the current literature on capacity investment by establishing a direct connection between the RCPs adopted by policymakers and firms' conventional capacity investment decisions. Although there is extensive research on conventional capacity investment, the link between RCPs and conventional capacity investment has yet to be explored. For instance, several studies examine capacity investment incentives and how those incentives decrease with higher market competition (e.g., [8]; [9]; [10]). Furthermore, the characteristics of the market and firms have been found to play a vital role in determining the conventional capacity investment chosen by firms ([11]; [12]; [13]). Other studies focus on factors that directly affect the profitability of the conventional capacity, such as the ability to exercise market power during peak hours ([14]), the volatility of input costs ([15]), or the relative impacts compared to renewable capacity ([16]). However, further investigation is warranted as the ongoing shift towards renewable energy poses new challenges, such as the possible indirect effects of RCPs.

In this context, the transition towards renewable energy sources has been shown to impact not only electricity prices and outputs ([17]; [18]) but also the decisions of firms regarding their investments in conventional capacity. For instance, using a two-stage optimization model, [19] examines the optimal investment mix in renewable and conventional energy capacity under uncertain input prices. Their findings suggest that as solar energy capacity increases, the need for conventional capacity during peak hours decreases, but this also increases the average market price and price volatility. Similarly, [20] develops a theoretical model to investigate the impact of renewable energy on firms' incentives to invest in conventional energy sources. They incorporate uncertainty in demand and supply (i.e., supply scenarios with and without wind) and find that introducing renewables leads to lower equilibrium prices and a decrease in conventional capacity investment.

The studies mentioned above assume that renewable output is compensated by market prices. However, many jurisdictions encourage the adoption of renewable energy sources through compensation mechanisms, such as FiT and FiP ([2]). Previous studies have analyzed these mechanisms and their impact on market outcomes, such as price, output, and carbon dioxide emissions ([21]; [22]; [23]; [18]). However, this paper advances the literature by examining how these RCPs affect firms' conventional capacity investment decisions. To the best of my knowledge, this study is the first to directly analyze this relationship.

**Figure 1:**
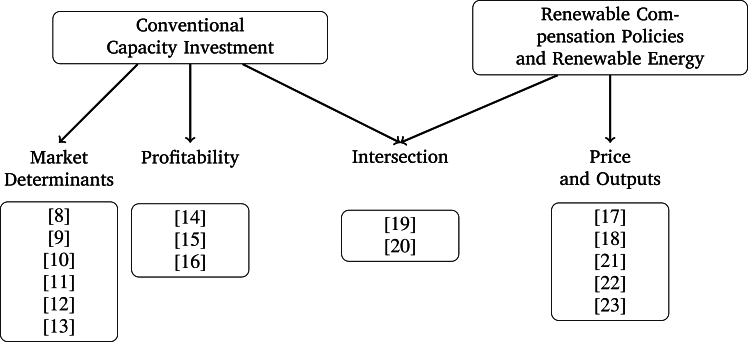
Literature Summary.

Fig. 1 summarizes the relevant literature for this paper divided into two main categories: conventional capacity investment decisions and RCPs and renewable energy. At the intersection of these two categories lie the two more related studies ([19] and [20]) that use market prices (instead of RCPs) to compensate renewable energy output. It is at this intersection where this paper brings its main contribution, developing a theoretical framework to analyze the effects of RCPs on conventional capacity investment decisions.

## 3 Model

In the model, two firms compete to supply a homogeneous good (i.e., electricity). In this game, firms make decisions in two stages: in the first stage, each firm i=1,2 simultaneously and independently decides its level of investment in conventional capacity, $K_{i}$, that is fixed and irreversible for the rest of the game. At this stage, the realization of demand is unknown, but the distribution of the demand function is common knowledge to both firms. In the second stage, taking $K_{i}$, the realization of the spot market demand, and the renewable capacity $R_{i}$ owned by each firm as given, firms simultaneously and independently engage in Cournot competition to supply electricity in the spot market. Consistent with previous studies, the analysis relies on Cournot competition to model the dynamics of a wholesale electricity market with strategic firms ([19]; [9]; [24]; [20]).

The spot market demand is denoted by the inverse linear demand curve P(Q)=a−βQ, where *a* and *β* are positive parameters. Firms face demand uncertainty characterized by the parameter *a*, which follows a uniform distribution $a∼U\left[\underline{a},\bar{a}\right]$, and this distribution is common knowledge for both firms. For analytical tractability, β=1, but the main conclusions are robust to this simplification. The total quantity supplied in the spot market is defined as $Q=q_{1}+q_{2}+\alpha (R_{1}+R_{2})$, where $q_{1}$ and $q_{2}$ represent the conventional outputs of firm 1 and 2, respectively, $R_{1}$ and $R_{2}$ represent the exogenous renewable capacity owned by each firm, and *α* represents the capacity utilization of the renewable generation.^3^ Throughout the paper it is assumed that α=1, but the results are robust to different values of *α*. For expositional purposes, a single spot market with different possible demand realizations is assumed (i.e., low, medium, and high demand), but the main conclusions hold if the model is extended to multiple spot market periods.

The cost function for the conventional generation of firm *i*, given capacity $K_{i}$, is given by $C_{i}(q_{i})=c_{i}q_{i}$ for $q_{i}\leq K_{i}$, where $c_{i}>0$, and $C_{i}(q_{i})=\infty$ for $q_{i}>K_{i}$. This last assumption restricts firms' conventional output to or below their maximum capacity. Additionally, the investment cost per MW of new conventional capacity is given by $C_{i}^{K}(K_{i})=\omega_{i}K_{i}$, where $\omega_{i}$ is a positive constant. For simplicity and following previous studies ([12]; [19]), it is assumed that firms have symmetric constant marginal cost for conventional generation and capacity investment (i.e., $c_{i}=c_{j}=c$ and $\omega_{i}=\omega_{j}=\omega$, with $\omega <\frac{\bar{a}-\underline{a}}{2}$).^4^ Renewable energy output is assumed to have zero marginal cost and is always dispatched; however, in equilibrium, renewable output does not fully satisfy total demand. Therefore, the market always requires a positive amount of conventional output to meet total spot market demand in equilibrium.^5^

Conventional generation is compensated at the spot market price. Renewable output is compensated either by a FiT, represented by ${\bar{P}}_{i}$ per MWh of output, or by a FiP that pays the firm the market price, P(Q), plus a premium, $m_{i}$, per MWh of output. This means that the total price per MWh of renewable output received by the firms under a FiP is $P(Q)+m_{i}$. Additionally, *δ* ∈ [0,1] is defined as the proportion of renewable output compensated by a FiT, and (1−δ) ∈ [0,1] as the proportion of renewable output compensated by a FiP. Both firms face the same *δ*, meaning they have the same fraction of their renewable output compensated by each RCP. This assumption is based on our interest in analyzing how changes in renewable compensation at the market level affect conventional capacity investment decisions. Analyzing changes in renewable compensation at the market level excludes the possibility of having a different *δ* for each firm, which is outside of the scope of this paper. Further, renewable CPs are often defined at the market rather than the firm level.^6^

**Figure 2:**
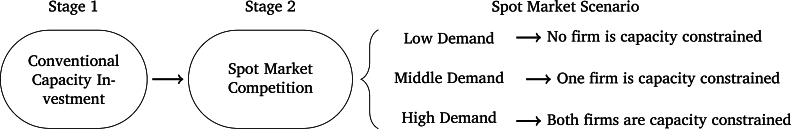
Model Summary.

Throughout the analysis, it is assumed that firms are risk neutral and have complete market information (i.e., firms know their rival's profit functions, capacity choices, cost structure, and the distribution of demand realization). The model relies on backward induction to solve for the Subgame Perfect Nash Equilibrium (SPNE). Fig. 2 provides an overview of the model's stages along with their key features. In the initial stage, firms determine their investments in conventional capacity. Subsequently, during the second stage, these firms engage in Cournot competition in the spot market to supply electricity. The spot market exhibits three possible scenarios of electricity demand realization: low, wherein firms produce below their capacities; medium, with one firm operating below capacity and the other at full capacity; and high, where both firms are operating at their maximum capacities.

## 4 Equilibrium analysis

This section starts by characterizing the SPNE of the game using backward induction, starting with the second stage (spot market). After deriving the equilibrium outcomes in the spot market, we move to the first stage to characterize the optimal capacity investment decision. In this first stage, each firm maximizes its expected profit function by considering three possible demand realizations: low, medium, and high. This section finishes with a discussion regarding the effects of the RCPs on the equilibrium capacity investment and other relevant market outcomes.

### 4.1 Spot market equilibrium

In this stage, we delve into the equilibrium outcomes assuming that, as the market demand function shifts outward, firm *i* is capacity constrained before firm *j*, where i,j=1,2, with i≠j (i.e., firm *i* optimal output reaches its maximum capacity before firm *j*). Note that with this notation, the analysis includes both cases when firm one is capacity-constrained first and when firm two is capacity-constrained first. This allows us to include all possible scenarios in the analysis. At this stage, firms take the capacity investments, $K_{i}$, and the realization of demand as given and face three possible cases based on the realization of the demand parameter *a*: low demand (i.e., low values of *a*), when neither firm is capacity constrained; medium demand (i.e., intermediate values of *a*), when firm *i* is capacity constrained and firm *j* is not; and high demand (i.e., high values of *a*), when both firms are capacity constrained.

#### 4.1.1 Low demand: unconstrained firms

In this case, the demand parameter *a* is sufficiently low, so neither firm is capacity constrained. Firms i,j=1,2, with i≠j, aim to maximize their spot market profits according to the following spot profit function^7^:

(1) $$\pi_{i}^{u}(q_{i}^{u},q_{j}^{u})=\left[P(Q)-c\right]q_{i}^{u}+{\bar{P}}_{i}\delta R_{i}+(P(Q)+m_{i})(1-\delta )R_{i},$$

where $q_{i}^{u}$ and $q_{j}^{u}$ represent the unconstrained conventional output for firms *i* and *j*, which are compensated at the spot price P(Q). Additionally, a fraction *δ* ∈ [0,1] of the renewable energy output $R_{i}$ is compensated at a fixed price ${\bar{P}}_{i}$, while the remaining fraction (1−δ) ∈ [0,1] is compensated at the spot price plus the premium $m_{i}$.

Solving the corresponding first-order conditions and focusing on interior solutions, the equilibrium outputs (equation (2)), prices (equation (3)), and profit functions (equations (4) and (5)) for the unconstrained case for firms i,j=1,2, with i≠j are as follows:

(2) $$q_{i}^{u⁎}=\frac{a+\delta (2R_{i}-R_{j})-3R_{i}-c}{3}\;\;;\;\;q_{j}^{u⁎}=\frac{a+\delta (2R_{j}-R_{i})-3R_{j}-c}{3}$$

(3) $$P^{u⁎}=\frac{a+2c-\delta (R_{i}+R_{j})}{3}$$

(4) $$\pi_{i}^{u⁎}=(P^{u⁎}-c)q_{i}^{u⁎}+\bar{P_{i}}\delta R_{i}+(P^{u⁎}+m_{i})(1-\delta )R_{i}$$

(5) $$\pi_{j}^{u⁎}=(P^{u⁎}-c)q_{j}^{u⁎}+\bar{P_{j}}\delta R_{j}+(P^{u⁎}+m_{j})(1-\delta )R_{j}$$

To ensure that firms produce positive amounts of conventional output, it is assumed that the lowest demand realization, net from renewable capacity for a given *δ*, is sufficiently high to offset the marginal cost of conventional generation (i.e., $\underline{a}+\delta (2R_{i}-R_{j})-3R_{i}>c$ for i,j=1,2, with i≠j).

In this unconstrained case, firm *i*'s equilibrium output of equation (2) is used to determine the highest value of *a* at which firm *i* is not capacity constrained (recall, firm *i* represents the firm that is constrained first). This value is denoted by $a_{i}$, where $\underline{a}\leq a_{i}<\bar{a}$:

(6) $$q_{i}^{u⁎}\leq K_{i}\;\;\;\;\;\Leftrightarrow \;\;\;\;\;a_{i}=3K_{i}+3R_{i}+c-\delta (2R_{i}-R_{j})$$

If the realization of *a* is greater than $a_{i}$, such that firm *j* still produces below its maximum capacity, we are in the medium demand case where firm *i* is capacity constrained and firm *j* is not.

#### 4.1.2 Medium demand: only firm *i* is capacity constrained

Following equation (6), a medium demand realization is defined to reflect a setting where firm *i* is capacity constrained in equilibrium (i.e., $a>a_{i}$), but its rival is not. In this case, the equilibrium output of firm *i* is $q_{i}^{K_{i}⁎}=K_{i}$, where the subscript represents the firm that is constrained first. In this case firm *i* reaches its maximum capacity $K_{i}$. Given that firm *j* is not capacity constrained, equation (1) for i,j=1,2, with i≠j becomes^8^:

(7) $$\pi_{i}^{K_{i}}(K_{i},q_{j}^{K_{i}})=\left[P(Q)-c\right]K_{i}+{\bar{P}}_{i}\delta R_{i}+(P(Q)+m_{i})(1-\delta )R_{i}$$

(8) $$\pi_{j}^{K_{i}}(K_{i},q_{j}^{K_{i}})=\left[P(Q)-c\right]q_{j}^{K_{i}}+{\bar{P}}_{j}\delta R_{j}+(P(Q)+m_{j})(1-\delta )R_{j},$$

where equations (7) and (8) represent the spot profit function for firms *i* and *j*, respectively, assuming that $q_{j}^{K_{i}}$ is such that firm *i*'s best response is $K_{i}$.

Solving the corresponding first-order conditions yields the following equilibrium outputs, price, and profit functions:

(9) $$q_{i}^{K_{i}⁎}=K_{i}\;\;;\;\;q_{j}^{K_{i}⁎}=\frac{a-K_{i}-R_{i}-2R_{j}-c+\delta R_{j}}{2}$$

(10) $$P^{K_{i}⁎}=\frac{a-K_{i}+c-R_{i}-\delta R_{j}}{2}$$

(11) $$\pi_{i}^{K_{i}⁎}=(P^{K_{i}⁎}-c)K_{i}+\bar{P_{i}}\delta R_{i}+(P^{K_{i}⁎}+m_{i})(1-\delta )R_{i}$$

(12) $$\pi_{j}^{K_{i}⁎}=(P^{K_{i}⁎}-c)q_{j}^{K_{i}⁎}+\bar{P_{j}}\delta R_{j}+(P^{K_{i}⁎}+m_{j})(1-\delta )R_{j}$$

Note that, in this case, the spot output and profits depend on whether firm 1 or 2 is capacity constrained first.

Now suppose that there exists an $a_{j}$, where $a_{i}<a_{j}\leq \bar{a}$ that represents the minimum value of *a* where firm *j* is capacity constrained in equilibrium. This means that for any $a_{i}\leq a<a_{j}$ firm *i* is capacity constrained and firm *j* is not, and for any $a_{j}\leq a\leq \bar{a}$, both firms are capacity constrained. Using the equilibrium spot output of firm *j* in equation (9), $a_{j}$ is defined in equation (13) as follows:

(13) $$q_{j}^{K_{i}⁎}\leq K_{j}\;\;\;\;\;\Leftrightarrow \;\;\;\;\;a_{j}=2K_{j}+K_{i}+R_{i}+2R_{j}+c-\delta R_{j},$$

which provides the highest value of *a* where firm *j* is not capacity constrained. If the realization of *a* is greater than $a_{j}$, then we are in the case where both firms are capacity constrained.

#### 4.1.3 High demand: both firms are capacity constrained

If the realization of *a* falls between $a_{j}$ and $\bar{a}$, we are in the high-demand scenario where both firms are capacity constrained in equilibrium (denoted by the upper subscript *c*), and their optimal output choices are equal to their capacities (i.e., $q_{i}^{c⁎}=K_{i}$ and $q_{j}^{c⁎}=K_{j}$ for i,j=1,2, with i≠j). In this case, the spot price is $P^{c⁎}=a-K_{i}-K_{j}-R_{i}-R_{j}$ and their profit functions are $\pi_{i}^{c⁎}=(P^{c⁎}-c)K_{i}+{\bar{P}}_{i}\delta R_{i}+(P^{c⁎}+m_{i})(1-\delta )R_{i}$ and $\pi_{j}^{c⁎}=(P^{c⁎}-c)K_{j}+{\bar{P}}_{j}\delta R_{j}+(P^{c⁎}+m_{j})(1-\delta )R_{j}$ ∀ i,j=1,2, with i≠j. These results are independent of whether firm 1 or 2 is capacity constrained first. Lemma 1 summarizes the spot market equilibrium structure derived in this section for each demand realization.

Lemma 1 *In the spot market, for given capacities and demand realization, the equilibrium structure is characterized as follows:*

1. *Low Demand: If*$\underline{a}\leq a<a_{i}$*, firms are not constrained by their capacities and sell their conventional output*$q_{i}^{u⁎}$*and*$q_{j}^{u⁎}$*at price*$P^{u⁎}$*.*
2. *Medium demand: If*$a_{i}\leq a<a_{j}$*, firm i is capacity constrained and firm j is not. In this firm i sells all its conventional capacity,*$K_{i}$*, and firm j sells*$q_{j}^{K_{i}⁎}$*at price*$P^{K_{i}⁎}$
3. *High Demand: If*$a_{j}\leq a\leq \bar{a}$*, both firms are capacity constrained so they sell all their conventional capacity,*$K_{i}$*and*$K_{j}$*, at price*$P^{c⁎}$*.*

*Where*$a_{i}=3K_{i}+3R_{i}+c-\delta (2R_{i}-R_{j})$*and*$a_{j}=2K_{j}+K_{i}+R_{i}+2R_{j}+c-\delta R_{j}$*.*

### 4.2 Capacity investment equilibrium

Once the equilibrium outcomes in the spot market have been derived for each possible demand realization, we proceed to the first stage of the model. In this stage, firms simultaneously and independently choose their conventional capacity investments, considering how these capacities will affect the equilibrium in the spot market. Given that the firms' expected profits depend on what firm is capacity constrained first, the notation is switched to express the capacity investment problem in terms of firms one and two. Firm one's expected profits, as a function of $K_{1}$ and $K_{2}$ for the cases when firm one is capacity constrained first (i.e., $a_{1}=3K_{1}+3R_{1}+c-\delta (2R_{1}-R_{2})<a_{2}=3K_{2}+3R_{2}+c-\delta (2R_{2}-R_{1})$) and when firm two is capacity constrained first (i.e., $a_{2}=3K_{2}+3R_{2}+c-\delta (2R_{2}-R_{1})<a_{1}=3K_{1}+3R_{1}+c-\delta (2R_{1}-R_{2})$), are given by^9^:(14)
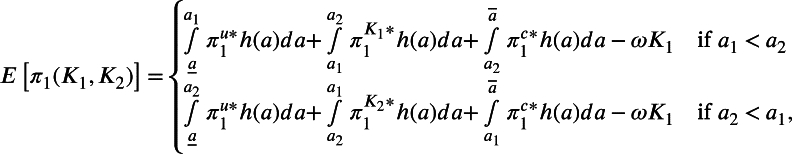
 where the equilibrium profits come from the spot market equilibrium structure illustrated in Lemma 1 and h(a) represents the probability density function of the uniform distribution of *a* (i.e., $h(a)=\frac{1}{\bar{a}-\underline{a}}$). Employing the Leibniz Rule to differentiate equation (14) and rearranging terms, the following first-order conditions for firm one are obtained assuming $a_{1}<a_{2}$ (note that the final result is analogous if the second case where $a_{2}<a_{1}$ is considered)^10^:(15)
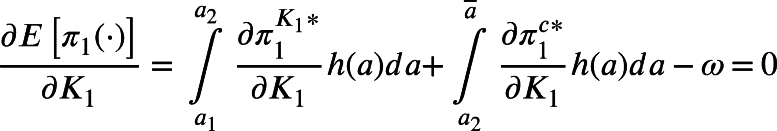
(16)
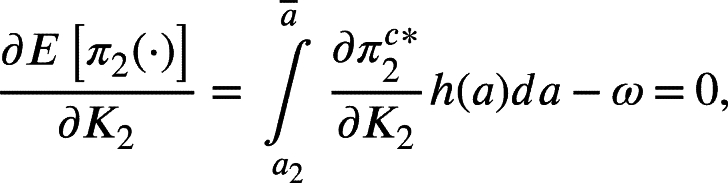
 where equation (15) represents the equilibrium condition for the firm that gets capacity constrained when realized demand is medium (in this case, firm one), and equation (16) represents the equilibrium condition for the firm that gets capacity constrained only with the high demand realization (in this case firm two).

From equations (15) and (16), firms will invest in capacity until their marginal benefit equals its marginal cost *ω*. The marginal benefit of the capacity is driven by the cases when capacity is binding. For firm one, this means cases with medium and high demand, while for firm two, only the case of high demand. The marginal unit of capacity invested has two opposing effects on profits when capacities are binding. To illustrate, consider the medium demand case for firm one where it can be shown that the derivative of firm one's realized profit is defined by:

(17) $$\frac{\partial \pi_{1}^{K_{1}⁎}}{\partial K_{1}}=\frac{\partial P^{K_{1}⁎}}{\partial K_{1}}\left[K_{1}+(1-\delta )R_{1}\right]+P^{K_{1}⁎}-c=0$$

The term $\frac{\partial P^{K_{1}⁎}}{\partial K_{1}}\left[K_{1}+(1-\delta )R_{1}\right]$ represents the disincentive of capacity investment: as capacity expands, the marginal profit of firm one decreases because it puts downward pressure on the spot price (note that $\frac{\partial P^{K_{1}⁎}}{\partial K_{1}}<0$). Notably, as *δ* approaches one (i.e., FiT dominates) in equation (17), there is less renewable output exposed to the spot market price, which decreases the disincentive effect of the capacity expansion, thereby strengthening capacity expansion incentives. However, the second term of equation (17), $P^{K_{1}}$, depends negatively on *δ*,^11^ which decreases the capacity expansion incentives, creating a trade-off that will be further investigated in subsection 4.3. Note that in the case when firm one is capacity constrained and firm two is not, $\frac{\partial \pi_{2}^{K_{1}⁎}}{\partial K_{2}}=0$. Further, the trade-off expressed in equation (17) also arises in the region of $a>a_{j}$. This is because $\frac{\partial \pi_{1}^{c⁎}}{\partial K_{1}}=\frac{\partial P^{c⁎}}{\partial K_{1}}\left[K_{1}+(1-\delta )R_{1}\right]+P^{c⁎}-c=0$, which contains the same negative and positive effects of *δ*.

Solving equations (15) and (16) with respect to $K_{1}$ and $K_{2}$ and checking the second-order conditions yields the optimal capacity investment for each firm.^12^ Lemma 2 presents the optimal capacity investment solution for firms one and two.

Lemma 2 *In the conventional capacity investment stage, there is a unique solution for the equilibrium conventional capacity investment independent of what firm is capacity constrained first. Each firm chooses its equilibrium conventional capacity investment, denoted by*$K_{1}^{⁎}$*and*$K_{2}^{⁎}$*, following equations*(18)*and*(19)^13^*:*

(18) $$K_{1}^{⁎}=\frac{\bar{a}-c-3R_{1}+\delta (2R_{1}-R_{2})-\sqrt{2\omega (\bar{a}-\underline{a})}}{3}$$

(19) $$K_{2}^{⁎}=\frac{\bar{a}-c-3R_{2}+\delta (2R_{2}-R_{1})-\sqrt{2\omega (\bar{a}-\underline{a})}}{3}$$

Note that the equilibrium conventional capacity investment described in Lemma 2 is independent of what firm is capacity constrained first. This is because firms are assumed to be symmetric in their cost structure (for conventional output and capacity investment), and the results only depend on the relative size of their renewable capacity.

### 4.3 Impacts of the RCPs

This section examines how RCPs impact investment incentives. First, we begin analyzing the spot market under the three possible demand realizations and the overall effect of *δ* on the expected equilibrium spot output and price. Subsequently, we move on to the capacity investment decision and how it is affected by *δ*.

#### 4.3.1 Spot market

Proposition 1 investigates how the equilibrium outcomes in the spot market are affected when the proportion of renewable output compensated by each RCP changes, holding capacities constant. Recall that the parameter of interest is represented by *δ* ∈ [0,1]. As *δ* approaches one (zero), renewable output is compensated by a FiT (FiP).

Proposition 1 *In the spot market stage, for each demand realization and firms*i,j=1,2*, with*i≠j*, as we vary the RCP used, the equilibrium output changes as follows:*

$$(i).\frac{\partial q_{i}^{u⁎}}{\partial \delta }=\left\{ \begin{array}{cc} >0 & if\;\;2R_{i}>R_{j}\\ =0 & if\;\;2R_{i}=R_{j}\\ <0 & if\;\;2R_{i}<R_{j} \end{array}\right.\;\;\;\;\;\;\;\;\;\;(ii).\frac{\partial q_{i}^{K_{i}⁎}}{\partial \delta }=0\;\;\;\;\;\;\;\;\;\;(iii).\frac{\partial q_{j}^{K_{i}⁎}}{\partial \delta }>0\;\;\;\;\;\;\;\;\;\;(iv).\frac{\partial q_{i}^{c⁎}}{\partial \delta }=\frac{\partial q_{j}^{c⁎}}{\partial \delta }=0$$

Proposition 1 summarizes the effects of varying the share of renewable output compensated by each RCP on the spot market equilibrium output for each demand realization. From (*i*), an increase in *δ* has an ambiguous effect on the unconstrained output, and it depends on the relative renewable capacity owned by each firm. If firm *i* owns a sufficiently large renewable capacity compared to its rival, then an increase in *δ* increases its spot market output; however, the opposite happens when firm *i* owns a sufficiently small renewable capacity relative to its rival (note that the analysis is analogous from the point of view of firm *j*). This effect can be understood using the opportunity cost intuition.

To the firm, the opportunity cost of selling an additional unit of conventional output means that it earns a lower price on the renewables it owns that are covered under a FiP. Now, when *δ* increases, the marginal cost of each firm (including the opportunity cost) decreases as well, but by different magnitudes depending on how much renewable capacity each firm has. This ambiguous effect is illustrated by the best response functions derived from equation (2).^14^ In equilibrium, changes in *δ* shift the best response functions of firms *i* and *j* in different magnitudes. For instance, suppose that $R_{i}$ is sufficiently large but $R_{j}$ is small, then as *δ* increases, the total amount of renewables subject to a FiP falls a lot for firm *i* but not much for firm *j*. As a result, with an increase in *δ*, the lost renewable revenues to firm *i* from an additional unit of conventional output become much less, while for firm *j*, it only falls by a small amount. Hence, as *δ* is increased, firm *i*'s best response function shifts out a lot, while firm *j*'s shifts out a little, potentially resulting in firm *i*'s output increasing while firm *j*'s output decreases.

Further, at the point when $2R_{i}=R_{j}$, the two channels offset each other for firm *i*, resulting in no change in its spot output with changes in *δ*. Note that when $2R_{i}=R_{j}$, it must be true that $2R_{j}>R_{i}$ (i.e., firm *j* owns a sufficiently large share of the total renewable capacity), so while firm *i* does not change its spot output because the two channels explained above offset each other, firm *j* increases its spot output. In this model, the point where $2R_{i}=R_{j}$ indicates the threshold for firm *i*'s renewable capacity to be considered sufficiently large or small. Therefore, owning a relatively large or small amount compared to its rival will determine the final effect of *δ* on the unconstrained spot output and, subsequently, on the conventional capacity investment.

Continuing with Proposition 1, when capacity is binding for firm *i*, changes in *δ* do not affect its spot market output, as indicated in (ii). This is because the firm has no incentives to decrease its production, and the firm wants to increase its production, but it is capacity constrained. On the other hand, for firm *j*, the effect is unambiguously positive according to (iii), meaning that an increase in the proportion of renewable output compensated by a FiT leads to an increase in firm *j*'s spot market output. This is because firm *i* does not react to the change in *δ*, limiting the strategic substitution of firm *j*'s output as $q_{j}^{K_{i}⁎}$ varies. Further, the pro-competitive impact of the FiT still exists, which increases firm *j*'s conventional generation output. Lastly, when firms are already producing at their maximum capacities, as stated in (iv), changes in *δ* do not affect the spot market outputs.

Next, Proposition 2 presents the effects of *δ* on the firm and market-level expected output and spot price.

Proposition 2 *In the spot market stage, for any demand realization and firms*i,j=1,2*, with*i≠j*, the firm and market-level expected output and expected price change as we vary the RCP used as follows:*

$$(i).\frac{\partial E(q_{i}^{⁎})}{\partial \delta }==\left\{ \begin{array}{cc} >0 & if\;\;2R_{i}>R_{j}\\ =0 & if\;\;2R_{i}=R_{j}\\ <0 & if\;\;2R_{i}<R_{j} \end{array}\right.\;\;\;\;\;(ii).\frac{\partial E(q_{mkt}^{⁎})}{\partial \delta }=\frac{R_{i}+R_{j}}{3}\;\;\;\;\;(ii).\frac{\partial E(P^{⁎})}{\partial \delta }=-\frac{R_{i}+R_{j}}{3}$$

Note that the results of (*i*) are analogous for firm *j*. From (*i*), the effect of *δ* on firm *i*'s expected spot market output is ambiguous and depends on the relative size of the renewable capacity owned by each firm. Similar to (*i*) in Proposition 1, when $2R_{i}>R_{j}$ ($2R_{i}<R_{j}$), the expected spot output of firm *i* increases (decreases) as *δ* increases, and it is unchanged when $2R_{i}=R_{j}$. While the effects of the individual firm's expected output are ambiguous, according to (ii), *δ* has an unambiguously positive effect on the expected total market output, $E(q_{mkt}^{⁎})$ (i.e., as delta approaches one total market output increases). Further, as shown in (iii), the effect of increasing *δ* on the expected spot price is unambiguously negative. This means that the spot price decreases as a FiT becomes more dominant in the market.

The results presented in propositions one and two have important societal implications and provide useful guidelines for policymakers. In particular, it is worth noting that while firm-level effects are ambiguous, overall market output increases and price decreases under a FiT and these effects depend on the relative size of the renewable energy capacity owned by the firms. This mean that policymakers seeking to minimize the price consumers pay for electricity should consider a FiT over a FiP. These results go in line with the literature that identify FiT as a promoter of competitiveness in the electricity markets, decreasing prices and increasing output ([22]; [18]); however, they must be cautiously considered because of the highly stylized nature of this model.

#### 4.3.2 Capacity investment

Based on Lemma 2, this section analyzes how changes in *δ* affect firms' incentives to invest in new conventional capacity. Proposition 3 summarizes the relationship between the optimal capacity investment and *δ*.

Proposition 3 *In the conventional capacity investment stage, for each demand realization and firms*i,j=1,2*, with*i≠j*, the equilibrium conventional capacity investment changes as we vary the RCP used as follows:*

$$\frac{\partial K_{i}^{⁎}}{\partial \delta }=\left\{ \begin{array}{cc} >0 & if\;\;2R_{i}>R_{j}\\ =0 & if\;\;2R_{i}=R_{j}\\ <0 & if\;\;2R_{i}<R_{j} \end{array}\right.$$

Proposition 3 shows that *δ* has an ambiguous effect on the equilibrium conventional capacity invested by the firms.^15^ Similar to the unconstrained case of Proposition 1, the effects of changing the proportion of renewable output paid a FiT and a FiP depend on the relative size of firms' renewable capacity.

First, suppose firm *i*'s renewable capacity is sufficiently large relative to its rival. In that case, an increase in the share of renewable output compensated by a FiT increases firm *i*'s incentives to invest in conventional capacity. As shown in Proposition 1, firm *i* has incentives to increase its conventional spot output. Therefore, increasing conventional capacity investment decreases the probability of being capacity constrained and allows firm *i* to increase its conventional output during scarcity periods when it is producing at capacity. Additionally, as *δ* approaches one (i.e., FiT dominates), there is less renewable output exposed to the spot market price, which decreases the disincentive effect of the capacity expansion, thereby strengthening capacity expansion incentives (first term of equation (17)). For sufficiently large renewable capacity owned by firm *i*, this effect dominates the negative impact on spot market price decreasing capacity expansion incentives (second term of equation (17)).

Second, when firm *i*'s renewable capacity is sufficiently small, increasing the proportion of renewable output compensated by a FiT decreases firm *i*'s conventional capacity investment incentives. Given that $R_{i}$ is sufficiently small, the positive effect described in the first term of equation (17) is relatively smaller than the negative effect on the market price that decreases conventional capacity investment incentives. Therefore, the net effect is a disincentive to invest in conventional capacity.

Similar to Proposition 1, at the point that $2R_{i}=R_{j}$, there is no change in firm *i*'s conventional capacity investment incentives with changes in *δ*. Note that when 2Ri=Rj, it must be true that 2Rj>Ri, so while firm *i* does not change its spot output, firm *j* increases it (recall the first case of Proposition 2). In this model, the point where 2Ri=Rj represents the threshold for firm *i*'s renewable capacity to be considered sufficiently large or small for the first positive effect to dominate or be dominated by the second negative effect of equation (17).

Note that, while the effect of *δ* on firm's equilibrium conventional capacity investments is ambiguous, the effect on overall equilibrium conventional capacity (i.e., the sum of both firms' conventional capacity, $K^{Market}$) is unambiguously positive. Proposition 4 summarizes the effect of *δ* on the overall equilibrium conventional capacity.

Proposition 4 *In the conventional capacity stage, for each demand realization and firms*i,j=1,2*, with*i≠j*, the equilibrium market conventional capacity investment changes as we vary the RCP used as follows:*

$$\frac{\partial K^{Market}}{\partial \delta }=\frac{R_{i}+R_{j}}{3}$$

Proposition 4 shows that *δ* has an unambiguously positive effect on conventional capacity investment at the market level. The magnitude of this effect depends on the renewable energy capacity owned by the firms. The result of Proposition 4 indicates that increasing the share of renewable output compensated by a FiT increases the overall conventional capacity investment in the market. As mentioned earlier, a FiT has a pro-competitive effect in the spot market, leading to more conventional output. As the market increases its conventional output, more conventional capacity is needed to allow more generation during high-demand hours when firms produce at maximum capacities.

The insights of propositions three and four capture the main takeaway of this study: a FiT has ambiguous effect on firm-level conventional capacity investment, but it has a positive effect at the market-level conventional capacity investment. These effects are in line with the expansion of natural gas generation facilities documented in [7], and support the pro-competitive effect of FiT described in [22] and [18]. Furthermore, the results show that the only relevant factor is the relative size of the renewable energy capacity owned by the firms. This information is readily available to policymakers who can use it to improve the design and implementation of the RCPs considered in this study; however, the results of this study are based on a duopoly model. As electricity markets are normally characterized by more than two firms, these results present a baseline, but a more thorough analysis is warranted.

## 5 Numerical example

This section provides a numerical example to illustrate the main results. While the model's parameters are calibrated to fit features of real-world electricity markets, it still represents a stylized model that simplifies reality. Therefore, the results are for illustrative purposes only.

First, assume that a∼U[635,905], the conventional capacity investment per MW is ω=8.9,^16^ and the marginal cost *c* is 70 based on a natural gas plant that generates during peak hours ([30]). The upper and lower bound for the demand parameter *a* were chosen to simplify the calculations and simulate the observed daily demand variation in jurisdictions like Texas and Alberta.^17^ Consistent with previous literature, these parameters yield a price elasticity of demand of 0.1 in the average perfectly competitive equilibrium without renewables ([31]).^18^

Following Proposition 3, the results are presented for the three cases when $2R_{1}⋚R_{2}$, assuming a scenario where most of the renewable output is compensated by a FiT (i.e., δ=0.8) and a scenario where most of the renewable output is compensated by a FiP (i.e., δ=0.2). In all cases, the total renewable energy capacity is set to 252 MW (i.e., $R_{1}+R_{2}=252$), which represents approximately 30% of the conventional output at the perfectly competitive equilibrium at the highest demand realization. For the case when $2R_{1}>R_{2}$, the renewable capacity is defined as $R_{1}=R_{2}=126$, while when $2R_{1}=R_{2}$, it is assumed $R_{1}=84$ and $R_{2}=168$. Finally, when $2R_{1}<R_{2}$, $R_{1}=52$ and $R_{2}=200$.

Table 1 presents the expected equilibrium market outcomes for each firm and scenario. Following [32], for the case of Germany in 2015, the renewable output compensated by a FiT is paid US$65 per MWh, while the premium above market price is US$5.^19^ The fixed price and premium values do not affect the total welfare analysis because they affect the consumer and producer surplus in opposing directions and the same magnitudes. However, these values will affect the distribution of consumer surplus and firms' profits.

**Table 1:** Numerical Example Results.

	$2R_{1}>R_{2}$		$2R_{1}=R_{2}$		$2R_{1}<R_{2}$	
	$R_{1}=R_{2}=126$		$R_{1}=84;R_{2}=168$		$R_{1}=52;R_{2}=200$	
Variable	*δ* = 0.2	*δ* = 0.8	*δ* = 0.2	*δ* = 0.8	*δ* = 0.2	*δ* = 0.8
*E*(*P*)	292.5	242.1	292.5	242.1	292.5	242.1
*E*(*q*_1_)	112.8	137.9	146.4	146.4	171.9	152.8
*E*(*q*_2_)	112.8	137.9	79.2	129.6	53.6	123.2
*K*_1_	137.6	162.8	171.2	171.2	196.8	177.6
*K*_2_	137.6	162.8	104.0	154.4	78.4	148.0
*K*^*Market*^	275.2	325.6	275.2	325.6	275.2	325.6
*E*(*CS*)	62,993	84,085	62,993	84,085	62,993	84,085
*E*(*π*_1_)	56,196	35,780	52,831	32,891	50,268	30,691
*E*(*π*_2_)	56,196	35,780	59,561	38,669	62,125	40,870
*E*(*TS*)	175,387	155,645	175,387	155,645	175,387	155,645

E(CS) and E(TS) denote expected consumer and expected total surplus, respectively.						

Table 1 illustrates the findings of Section 4.3. First, note that the expected equilibrium price, expected consumer and total surpluses, E(CS) and E(TS), remain constant as the renewable output allocation varies across the two firms for a given *δ* value. This is because the total renewable capacity (252 MW) is fixed for all cases, so the aggregated conventional capacity is always the same for a given *δ*. This restriction is imposed so that the results are comparable across the three cases. Further, note that TS does not depend on the levels of the fixed price, $\bar{P_{i}}$, and the premium $m_{i}$ because they enter with opposite signs into the CS and the firms' profits, canceling each other out. However, the fixed price and the premium values will affect the distribution of CS and firms' profits.

Following (*i*) of Proposition 1, firms have incentives to increase their expected equilibrium spot market output as *δ* increases when $2R_{i}>R_{j}$. In this first case, firm *j* also increases its output because $2R_{j}>R_{i}$.^20^ When $2R_{i}=R_{j}$, firm *i*'s spot output remains unchanged, while firm *j*'s increases, this is because if $2R_{i}=R_{j}$, it must be true that $R_{j}>R_{i}$, which is represented in the first case of Table 1. Since firm *j* owns more renewable capacity than its rival, it produces relatively less conventional output. This means that firm *j* has a smaller proportion of output exposed to the spot price reduction as it expands its output when *δ* increases, strengthening the incentives to expand its spot market output. Further, when $2R_{i}<R_{j}$, Table 1 shows the opposite effect for both firms as *δ* increases. Firm *i* reduces its spot market output in response to firm *j*'s aggressive spot-market output increase. Nevertheless, in line with Proposition 4, total conventional capacity unambiguously increases as *δ* increases.

Expected spot market price unambiguously decreases when *δ* increases, consistent with (ii) in Proposition 2. Independent of the relative renewable capacity ownership, a FiT decreases the equilibrium price suggesting lower incentives to exercise market power. The results of conventional capacity investments are consistent with Proposition 3. Firm *i* increases (decreases) its conventional capacity investment when $2R_{i}>R_{j}$ ($2R_{i}<R_{j}$), while it keeps it unchanged when $2R_{i}=R_{j}$. Firm *j* always increases its conventional capacity investment because in all three cases of Table 1, it is true that $2R_{j}>R_{i}$. Recall that increasing the conventional capacity investment gives the firm more flexibility to react to an increase in *δ* by expanding its conventional output during high-demand periods.

Finally, the spot price decrease always increases the expected *CS* when a FiT becomes more dominant. However, this increment in expected *CS* is offset by a reduction in firms' profits, leading to an overall reduction in expected *TS* as *δ* increases.

This numerical example illustrates how firms' behavior and incentives change as a response to changes in renewable output compensation. The findings are consistent with the results from Section 4.3, illustrating the ambiguous effect of RCPs on firm-level conventional capacity investment and the unambiguous positive effect of a FiT on the market-level conventional capacity investment. The model presents the first theoretical framework to quantify the direct relationship between RCPs and conventional energy investments. Further, the results highlight the main theoretical contribution of this study: the relative size of the renewable capacity owned by the firms is crucial in shaping their behavior.

## 6 Policy suggestions

As mentioned earlier, all the studies that focus on the relative investments incentives of conventional and renewable energy assume that renewable output is compensated by market prices. However, many jurisdictions encourage the adoption of renewable energy sources through compensation mechanisms, such as FiT and FiP ([2]). Therefore, this paper proposes a novel theoretical framework that links how these RCPs affect firms' conventional capacity investment decisions.

Admittedly, the theoretical results are based on a stylized model. Nevertheless, the model sheds light on the importance of understanding the spillover effects of RCPs on conventional renewable capacity. As jurisdictions increasingly employ these RCPs while advocating for conventional capacity investment to complement the renewable energy transition, it is important to be aware of the relationship between them. The model's main result suggests that jurisdictions relying on conventional capacity investment as a backup option for their renewable energy transition may favor FiTs over FiPs. This is because a FiT encourages the adoption of renewable energy sources, but also stimulates the investment in conventional generation capacity.

Additionally, the insights of the model reveal that RCPs affect the market-level outcomes unambiguously; however, their effects on individual firms depend on the level of renewable energy capacity owned by each firm. This highlights the importance of understanding the market structure and characteristics when designing and implementing a RCP. Policymakers should consider the possible winners and losers from their policies to better assess their possible impacts. This study suggests that existing renewable energy capacity plays a crucial role and must be taken into consideration.

Finally, the results from the numerical analysis suggest that the different RCPs not only affect firms' incentives to invest in conventional capacity, but also overall profits and consumer's surplus. In fact, a FiT unambiguously increases consumer's surplus, while decreasing firms' profits. In general, policymakers aim to maximize overall society surplus (i.e., consumer plus producer surplus), which may lead to different optimal RCP depending on the jurisdiction and the weights assigned by the regulator or policymaker to the consumers and the producers.

All in all, this study is a first step to provide additional tools to policymakers when designing and implementing the appropriate RCP for their jurisdiction. The model sheds light on the unintended effects of RCPs on conventional capacity investments and identifies the existing renewable energy capacity as the most important factor determining the these effects.

## 7 Conclusion

Despite the broad implementation of RCPs, little is known about their effects on the incentives to invest in conventional energy capacity. Many regions worldwide continue expanding their natural gas capacity alongside their efforts to adopt renewable energy sources. This paper derives a direct channel through which two different RCPs, feed-in tariff (FiT) and feed-in premium (FiP), and the investment in conventional capacity interact. The analysis relies on a two-stage duopoly model in a setting with imperfect competition and uncertain demand. In the first stage, firms simultaneously and independently decide their conventional capacity investment. In the second stage, taking conventional capacity investments, the realization of the spot market demand, and the renewable capacity own by each firm as given, firms engage in Cournot competition at the spot market to supply electricity.

In line with previous studies, the model shows that the expected spot price decreases as a FiT becomes more dominant. Further, increasing the proportion of renewable output compensated by a FiT increases the total capacity investment in the market. However, it has an ambiguous effect on each firm's conventional capacity investment level. The direction of this effect depends on the size of the renewable capacity owned by the firm relative to its rival. For instance, when a firm owns a sufficiently large (small) share of the renewable capacity, its expected conventional output is relatively smaller (larger) compared to its rival's. So, increasing the share of renewable output compensated by a FiT pushes the firm to behave more (less) competitively, expanding (contracting) its conventional spot output. This pro-competitive (anti-competitive) effect of the FiT is because less (more) of the firm's spot output is exposed to the reduction in the spot market price as its output expands. Additionally, the ability to expand the conventional spot output is determined by the conventional capacity owned by the firm. Therefore, when a firm owns a sufficiently large (small) share of the renewable capacity, it is incentivized to increase (decrease) its conventional capacity investment.

The illustrative numerical example shows that the expected consumer surplus unambiguously increases as more renewable output is compensated by a FiT, which can be explained by the decrease in the expected spot price. However, the increment in expected consumer surplus is offset by a sharper decrease in firms' profit, leading to a decrease in expected total surplus when a FiT becomes more dominant. The numerical example is calibrated to fit real-world electricity markets, but a more comprehensive empirical analysis is needed to study the robustness of the model's results.

While informative, the model presented has several limitations. In reality, the same jurisdiction may have RCPs applied differently to each firm, especially when analyzing an extended period. This paper assumes that the same proportion of renewables is compensated by a specific RCP (denoted by the parameter *δ*). Future research may relax this assumption and allow each firm to have its own renewable output compensation structure (i.e., different *δ* for each firm). The results are expected to still depend on the renewable capacity size, but formal proof is needed.

Additionally, the model assumes that renewable output is known by the firms when deciding their conventional capacity investment; however, in reality, the realization of renewable output may significantly impact the firms' investment decisions. Different renewable generation sources may affect conventional energy sources differently,^21^ so this area warrants further research.

Finally, renewable capacity is assumed to be exogenous for each firm. In reality, conventional and renewable energy capacity investment decisions are endogenous, increasing the importance of each RCP's fixed price and premium. This paper aims to isolate a single channel through which the compensation mechanism affects conventional investment decisions, so endogenizing the renewable capacity is out of the scope of this paper. However, future research may build upon this paper and other studies that allow endogenous renewable capacity investment to understand the dynamics in this more realistic setting.

## CRediT authorship contribution statement

**Boris Ortega Moreno:** Writing – review & editing, Writing – original draft, Formal analysis, Conceptualization.

## Declaration of Competing Interest

The authors declare that they have no known competing financial interests or personal relationships that could have appeared to influence the work reported in this paper.

## Data Availability

No data was used for the research described in the article. Therefore, no data has been deposited into any publicly available repository.

## Appendix A Derivation of capacity investment stage

In this stage, firms simultaneously and independently choose their capacity investment understanding how these capacities will affect the equilibrium in the spot market. For mathematical tractability, it is assumed that firm one is capacity constrained before firm two, but the results are independent of what firm is assumed to be capacity constrained first. Firm i=1,2 choose its capacity investment, $K_{i}$, to maximize its expected profits, represented by equation (14).

Define $f_{i}^{x}(K_{i},a_{t})$ for $x=u,K_{1},c$ (i.e., the three cases described in section 3) as the argument of each integral of equation (14) evaluated at *a*. Using the Leibniz rule to differentiate equation (14) for i=1,2, we obtain:

(20)

We know that $\frac{\partial \underline{a}}{\partial K_{i}}=\frac{\partial \bar{a}}{\partial K_{i}}=\frac{\partial f_{i}^{u}(K_{i},a)}{\partial K_{i}}=0$, which simplifies equation (20) to:

(21)

Further, for firm two's FOC we know that $\frac{\partial a_{1}}{\partial K_{2}}=\frac{\partial f_{2}^{K_{1}}(K_{2},a)}{\partial K_{2}}=0$. Rearranging terms the FOC's for firm one and two continue as follows:

(22)

(23)

Evaluating the arguments of the integrals at $a_{1}$ and $a_{2}$, we can eliminate the first and second terms of equation (22) and the first term of equation (23). This is because

$f_{1}^{u}(K_{1},a_{1})=f_{1}^{K_{1}}(K_{1},a_{1})=\left[(K_{1}+R_{1}(1-\delta ))K_{1}+\bar{P_{1}}\delta R_{1}+(K_{1}+c+R_{1}(1-\delta )+m_{1})(1-\delta )R_{1}\right]h(a)$ and

$f_{1}^{K_{1}}(K_{1},a_{1})=f_{1}^{c}(K_{1},a_{1})=\left[\left(K_{2}+R_{2}(1-\delta )\right)K_{1}+\bar{P_{1}}\delta R_{1}+(K_{2}+c+R_{2}(1-\delta )+m_{1})(1-\delta )R_{1}\right]h(a)$.

Solving the derivatives inside the integrals and rearranging terms, equations (22) and (23) become:

(24)

(25)

Indexing firms i,j=1,2, with i≠j, to represent all solutions and solving equations (24) and (25), we obtain:

(26) $$\frac{\partial E\left[\pi_{i}(\cdot )\right]}{\partial K_{i}}=\omega (\underline{a}-\bar{a})+\epsilon -\frac{{\left(c+3K_{i}+3R_{i}-\delta (2R_{i}-R_{j})\right)}^{2}}{4}+\frac{{\bar{a}}^{2}}{2}-\frac{\beta_{i}^{2}}{4}+\beta_{j}(\beta_{i}-\bar{a})=0$$

(27) $$\frac{\partial E\left[\pi_{j}(\cdot )\right]}{\partial K_{j}}=\omega (\underline{a}-\bar{a})+\frac{{\bar{a}}^{2}}{2}+\beta_{i}\left(\frac{\beta_{i}}{2}-\bar{a}\right)=0$$

where $\epsilon =\left(\beta_{j}-K_{j}+R_{j}(\delta -1)\right)\left(K_{i}-K_{j}+(1-\delta )(R_{i}-R_{j})\right)$ and $\beta_{i}=c+K_{i}+2K_{j}+R_{i}+R_{j}(2-\delta )$ and $\beta_{j}=c+K_{j}+2K_{i}+R_{j}+R_{i}(2-\delta )$ ∀ i,j=1,2, with i≠j.

Solving equations (26) and (27) with respect to $K_{i}$ and $K_{j}$ and checking the second-order conditions, yield the optimal capacity investment for each firm, where the only feasible solution is given by Proposition 3.

## Appendix B Proof of solution uniqueness

Based on equations (26) and (27), the model predicts four possible pair solutions for $K_{i}^{⁎}$ and $K_{j}^{⁎}$. Throughout the proof, each set of solutions is characterized by superscripts 1,2,3,4. This proof focuses on the case when firm one is capacity constrained before firm two because the proof is analogous to the other case. The following expression describes solutions one and two:

(28) $$K_{i}^{⁎1,2}=\frac{\bar{a}-c-3R_{i}+2\delta R_{i}-\delta R_{j}\pm \sqrt{2\omega (\bar{a}-\underline{a})}}{3}$$

Solutions three and four are as follows:

(29) $$K_{1}^{⁎3}=\bar{a}-c-R_{1}-\delta R_{2}+\sqrt{2\omega (\bar{a}-\underline{a})}\;\;;\;\;K_{2}^{⁎3}=R_{2}(\delta -1)-\sqrt{2\omega (\bar{a}-\underline{a})}$$

(30) $$K_{1}^{⁎4}=\bar{a}-c-R_{1}-\delta R_{2}-\sqrt{2\omega (\bar{a}-\underline{a})}\;\;;\;\;K_{2}^{⁎4}=R_{2}(\delta -1)+\sqrt{2\omega (\bar{a}-\underline{a})}$$

To prove the feasibility of the solutions, we use equations (6) and (13) that define $a_{i}$ and $a_{j}$, respectively, and the fact that, by definition, the realized level of demand, *a*, falls between $\underline{a}$ and $\bar{a}$.

First, let us start with solutions one and two. By definition $\underline{a}\leq a_{i}\leq \bar{a}$, and using equation (6) and substituting $K_{1}^{⁎1,2}$ from equation (28), we obtain:

(31) $$\underline{a}\leq 3\left[\frac{\bar{a}-c-3R_{1}+2\delta R_{1}-\delta R_{2}\pm \sqrt{2\omega (\bar{a}-\underline{a})}}{3}\right]+3R_{1}+c-\delta (2R_{1}-R_{2})\leq \bar{a}$$

(32) $$\underline{a}\leq \bar{a}\pm \sqrt{2\omega (\bar{a}-\underline{a})}\leq \bar{a}$$

Recall that $\omega (\bar{a}-\underline{a})>0$, so for equation (32) to be feasible, the sign of the squared root must be negative. This rules out $K_{1}^{⁎2}$ from the feasible solutions of the model. Note that employing the same analysis for $a_{j}$ with equation (13) yields the same conclusion of equation (32): only solution one is feasible. Further, note that equation (32) can be rearranged, using only solution one (i.e., negative squared root) as:

(33) $$\sqrt{2\omega (\bar{a}-\underline{a})}\leq \bar{a}-\underline{a}\leq \bar{a},$$

which is always true for $\omega <\frac{\bar{a}-\underline{a}}{2}$. This shows that solution one is always at or within the upper and lower bounds of the uniform distribution of *a*.

Regarding solution three, it is straightforward to notice that $K_{2}^{⁎3}$ is strictly negative, which is a contradiction because the capacity level investment is bounded below by zero. As *δ* ∈ [0,1], the maximum value that $K_{2}^{⁎3}$ can take is $-\sqrt{2\omega (\bar{a}-\underline{a})}$, which is strictly negative because $\omega (\bar{a}-\underline{a})>0$. This rules out solution three as part of the model solution.

Finally, solution four yields a value of $a_{j}$ greater than the upper bound $\bar{a}$. Using equation (13) and substituting the optimal capacity investment values of expression (30), we obtain:

(34) $$2\left[R_{2}(\delta -1)+\sqrt{2\omega (\bar{a}-\underline{a})}\right]+\bar{a}-c-R_{1}-\delta R_{2}-\sqrt{2\omega (\bar{a}-\underline{a})}+R_{1}+R_{2}(2-\delta )-c\leq \bar{a}$$

(35) $$\bar{a}+\sqrt{2\omega (\bar{a}-\underline{a})}\leq \bar{a}$$

As argued before, equation (35) leads to a contradiction given that, by definition, $\omega (\bar{a}-\underline{a})>0$. This proves that solution four is unfeasible and that the model yields a unique feasible solution. ■

## Footnotes

1 The simplest form of FiT is when the regulator provides the firm with a fixed price per unit of output. In the case of FiPs, the regulator can directly pay a premium above market price to the firm, or other forms of payment like tax credits.

2 The definition of renewable portfolio standards depends on the jurisdiction, but in general, they determine the minimum share of generation of consumption covered by renewable energy.

3 Contrary to [19], in which renewable energy capacity is endogenous, this paper focuses on the RCPs and their effect on conventional capacity investment. To better isolate these effects, renewable capacity is set exogenously. Future research may relax this assumption and endogenize renewable capacity investment.

4 Note that $\omega <\frac{\bar{a}-\underline{a}}{2}$ assumes that the capacity investment cost is positively related to demand uncertainty. As uncertainty increases (i.e., $(\bar{a}-\underline{a})⇀\infty$), the capacity investment cost increases. This assumption aligns with the evidence showing that firms facing higher demand uncertainty have a more rigid short-run cost structure with higher fixed and lower variable costs ([25]; [26]; [27]).

5 To ensure that firms produce positive amounts of conventional output, it is assumed that the lowest demand realization, net from renewable capacity for a given *δ*, is sufficiently high to offset the marginal cost of conventional generation (i.e., $\underline{a}-R_{i}(3-2\delta )-\delta R_{j}>c$ for i,j=1,2, with i≠j).

6 Refer to [28] for an RCP example in Alberta and [1] for a comparison among several jurisdictions across Europe and North America that employ different RCPs.

7 The analysis is analogous for firm *j*.

8 Note that the spot profit function to be maximized depends on whether the firm is constrained first. Hence, the two different spot profit functions.

9 Note that firm two's expected profits are analogous to equation (14), but replacing firm one's profit expressions for firm two's.

10 Refer to Appendix A for a detailed derivation of the capacity investment stage.

11 The equilibrium price when only firm one is capacity constrained is $P^{K_{1}⁎}=\frac{a-K_{1}-R_{1}+c-\delta R_{2}}{2}$, hence $\frac{\partial P^{K_{1}⁎}}{\partial \delta }=-\frac{R_{2}}{2}$.

12 Refer to Appendix B for the proof regarding solution feasibility.

13 Similar to Section 4.1.1, it is assumed that $\underline{a}-R_{i}(3-2\delta )-\delta R_{j}>c+\sqrt{2\omega (\bar{a}-\underline{a})}$ for i,j=1,2, with i≠j, which ensures that conventional capacity investments are never negative. Additionally, recall that $q_{i}^{u⁎}$ is always smaller than $K_{i}^{⁎}$ for any $a<a_{1}$, and it is equal to $K_{i}^{⁎}$ otherwise.

14 The best response function for firm *i* is $q_{i}^{u}(q_{j}^{u})=\frac{a_{t}-q_{j}^{u}-2R_{i}-R_{j}-c+\delta R_{i}}{2}$, which is analogous for firm *j*. The best response function shows that *δ* affects firms *i* equilibrium output through the effect on its rival's output, $q_{j}^{u}$, and directly through $\frac{R_{i}}{2}$.

15 Note that the results of Proposition 3 are analogous for $K_{j}$ when i,j=1,2, with i≠j.

16 According to [29], a combined-cycle natural-gas plant's weighted levelized capital cost is US$7.72 per MWh. Assuming a capacity factor of 87%, the weighted levelized capital cost per MW of capacity installed is US$8.9. Note that only the levelized capital cost of conventional capacity investment is included. Considering the total levelized cost of electricity increases *ω* to US$35.53 ([29]). Nevertheless, the main conclusions are robust to changes in the cost of capacity investment.

17 In Texas, the ratio of average load in the lowest to highest-demand hours for 2018 was 0.74 (estimated with data from the hourly load data archives of the Electric Reliability Council of Texas), while for Alberta was 0.67 (estimated with data from the Alberta Electric System Operator).

18 In a perfectly competitive equilibrium without renewable, the equilibrium price and market quantity are $70 and 700, respectively. Given the average demand function Q=770-P, the price elasticity of demand in equilibrium is $\eta =\frac{\partial Q}{\partial P}\frac{P}{Q}=0.1$.

19 [32] provides the fixed price and the premium in Euros. The average EUR-USD exchange rate in 2015 was 1.11.

20 Note that the symmetric equilibrium shown in Table 1 arises because $R_{i}=R_{j}$.

21 For example, [33] shows that renewables like wind energy tend to displace coal generation, while solar displaces natural gas.
